# Retracted: Rapidly increasing body mass index among children, adolescents and young adults in a transitioning population, South Africa, 2008–15

**DOI:** 10.1093/ije/dyy248

**Published:** 2018-10-29

**Authors:** B Sartorius, K Sartorius, M Taylor, J Aagaard-Hansen, N Dukhi, C Day, N Ndlovu, R Slotow, K Hofman

First published online: 14 December 2017, *Int J Epidemiol*, 2018, 47(3), 942–952, doi: https://doi.org/10.1093/ije/dyx263.

The above article from *International Journal of Epidemiology* ‘Rapidly increasing body mass index among children, adolescents and young adults in a transitioning population, South Africa, 2008–15’ by B Sartorius, K Sartorius, M Taylor, J Aagaard-Hansen, N Dukhi, C Day, N Ndlovu, R Slotow and K Hofman published online on 14 December 2017 and in the *International Journal of Epidemiology*, Volume 47, Issue 3, June 2018, Pages 942–952 by Oxford University Press has been retracted by agreement between the journal Editor, Professor Stephen Leeder, the lead author Professor Benn Sartorius and all co-authors, the International Epidemiological Association and Oxford University Press.

The retraction follows the discovery of a mistake relating to the analysis and interpretation of the South African National Income Dynamics Study (SA-NIDS) data, especially that from Wave 4 presented in Figure 1. This error resulted from not incrementing age between Waves 1 and 4 and so not accounting for the normal increase with age in BMI among young people. Recalculation of the estimates shows that there is little change in mean BMI by age over the period between Waves 1 and 4 and thus no evidence of the large increase in the prevalence of overweight and obesity in young people shown in Figure 3 that is the main finding of the study. All authors accept responsibility and apologise for this error.

